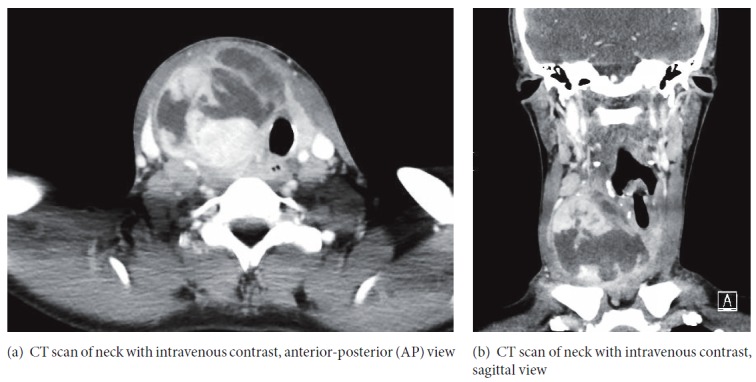# Methicillin-Sensitive* Staphylococcus aureus* Suppurative Thyroiditis with Thyrotoxicosis

**DOI:** 10.1155/2017/4018193

**Published:** 2017-12-19

**Authors:** Sha Yan, Laryssa Patti

**Affiliations:** Department of Emergency Medicine, Rutgers Robert Wood Johnson Medical School, 1 RWJ Place, MEB 104, New Brunswick, NJ 08901, USA

## Abstract

Intravenous drug use (IVDU) can lead to numerous complications from skin abscesses to bacteremia to endocarditis. Here, we present a rare case of acute suppurative thyroiditis as a complication of IVDU, in which a 26-year-old female with a past medical history of IVDU presented to the emergency department for evaluation of a large right sided neck mass. On exam, she had signs of sepsis and thyrotoxicosis. Fine needle aspiration confirmed suppurative thyroiditis. Blood cultures and culture from fine needle aspiration grew* Staphylococcus aureus*. Patient was treated with 2 weeks of intravenous antibiotics with good resolution of her symptoms.

## 1. Introduction

Acute suppurative thyroiditis (AST) is a rare disease as the thyroid is an uncommon place for infection. Most cases are seen in children with congenital pyriform sinus fistulae that allow for infection to spread to thyroid gland. Here, we report a case of a young adult female who presented with AST as a suspected complication of intravenous drug abuse.

## 2. Case

The patient is a 26-year-old female with past medical history of intravenous drug use (IVDU) who presented to the emergency department (ED) complaining of increasing neck mass and sore throat that worsened over the past few days. She described neck swelling for over a year that was evaluated by ultrasound at an outside hospital; the patient did not know the hospital or the result. She reported an acute worsening of neck swelling associated with a sore throat. She otherwise denied fever, cough, rhinorrhea, dysphagia, or dyspnea. Of note, patient reported injection of heroin to her right forearm two days prior to arrival. She reported smoking cigarettes but denied alcohol or other illicit drug use.

**Figure 1:**
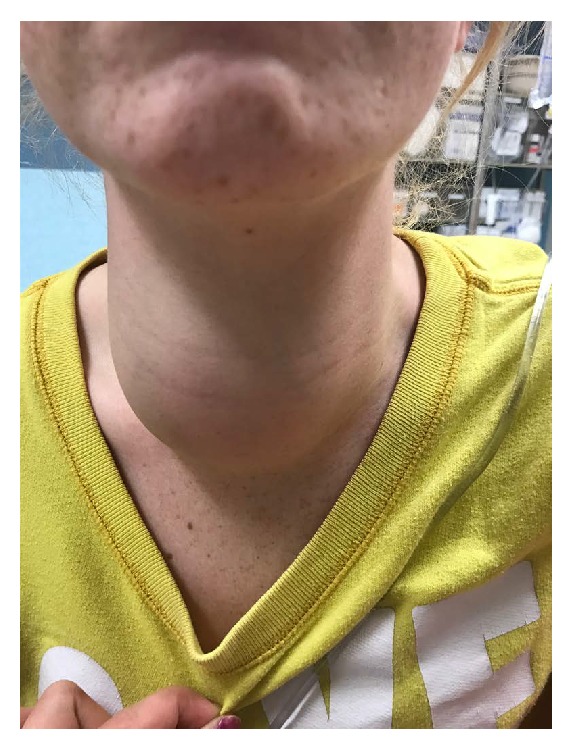
Anterior neck mass on the day of presentation.

While she reported no fever at home, upon triage, she had an oral temperature of 38.3°C. The remainder of her triage vital signs revealed tachycardia to 130 beats per minute (bpm), respiratory rate of 16 breaths per minute, blood pressure 134/88 mmHg, and 99% oxygen saturation on room air. She appeared in no acute distress and was breathing comfortably, speaking in full sentences, and managing her secretions. On examination, she had a large, nontender right sided neck mass nearly 10 × 6 cm (Figure 1). She was able to range her neck without difficulty. Oropharyngeal exam revealed mild erythema of the posterior pharynx without exudate. Lungs sounds were clear to auscultation, and heart sounds were tachycardic but regular and without murmur. She was found to have two 2 cm × 2 cm abscesses on her right forearm. The remainder of her exam was within normal limits.

Laboratory results showed white blood cell count 23.8 × 10^3^/uL, hemoglobin 9.8 g/dL, platelet count 608 × 10^3^/uL, sodium 127 mmol/L, potassium 3.5 mEq/L, chloride 86 mmol/L, thyroid stimulating hormone 0.02 mIU/L, free T3 5.1 pg/ml, and free T4 3.89 ng/dL. The remainder of her chemistry was within normal limits. CT of soft tissue neck with intravenous contrast showed 6.4 × 6.1 × 7.1 cm heterogeneous mass with associated mild-to-moderate edema and inflammatory changes as well as mass effect on the trachea, right internal jugular, and right common carotid artery (Figures 2(a) and 2(b)).

The patient was treated with acetaminophen 1 gram PO for fever, 30 cc/kg bolus of normal saline for tachycardia, and ampicillin/sulbactam 3 g IV empirically as treatment for suspected cellulitis and pharyngitis. Blood cultures were drawn. Her right forearm abscesses were incised and drained of purulent material and wound packing was inserted. Upon reassessment after completion of normal saline bolus, patient had temperature 36.7°C, blood pressure 132/66 mmHg, heart rate 92 bpm, respiratory rate 14, and oxygen saturation of 98% on room air.

During her hospital stay, she was started on oral naproxen and propranolol for the thyrotoxicosis. Her urine drug screen was positive for tetrahydrocannabinol (THC). Thyroid ultrasound revealed a large heterogenous necrotic hypervascular mass. Biopsy via fine needle aspiration (FNA) showed acute suppurative thyroiditis with focal follicular cell atypia. Culture from fine needle aspiration grew* Staphylococcus aureus*. One out of two blood cultures grew methicillin-sensitive* Staphylococcus aureus*. Given her FNA results and multiple skin abscesses, she was treated for bacteremia with intravenous antibiotics. Transesophageal echocardiography showed no vegetation on the heart valves. Human immunodeficiency virus (HIV) titers were negative, but she tested positive for hepatitis C. This patient received two weeks of intravenous antibiotic with resolution of leukocytosis and subsequently was discharged home on amoxicillin-clavulanic acid, naproxen, and propranolol.

## 3. Discussion

Acute suppurative thyroiditis (AST) is a rare condition, accounting for 0.1–0.7% of all thyroid disease [1]. The thyroid gland is an uncommon location for infection due to its large vascular supply, extensive lymphatic drainage, high iodine concentration, and anatomical encapsulation [2]. Up to 92% of cases of AST are found in children and young adults, and the majority of these have anatomical variants, such as congenital pyriform sinus fistula and a remnant third or fourth brachial fistula [3]. Therefore, ATS often presents on the left side, due to the atrophic right ultimo brachial body [1]. Moreover, AST usually occurs in those who have underlying thyroid pathology, such as neoplastic thyroid nodules, Hashimoto's thyroiditis, subacute thyroiditis, multinodular goiters, or penetrating trauma to the thyroid gland [2]. Cases reported in older adults were associated with degrees of immunocompromisation, such as infection with HIV [4], renal failure requiring hemodialysis [5], or reception of stem cell transplant [6]. Symptoms of AST include fever, dysphagia, and tender neck mass. It is often preceded by an upper respiratory infection [2]. The most common organisms associated with AST are* Staphylococcus aureus*,* Streptococcus* species,* Escherichia coli*,* Clostridium* species, Gram-negative bacilli, and anaerobes. Other fungal and parasitic organisms have also been reported in immunocompromised patients [1]. Thyroid functions in these patients are usually normal [2]. However, thyrotoxicosis has been reported when the destruction of the gland causes the release of thyroid hormone into the circulation [1].

In this patient, previous history of thyroid disease was unclear. While she reported chronic neck mass, she did not know the results or location of her previous work-up. A congenital pyriform fistula is unlikely in this patient due the age of onset and her predominantly right sided neck mass. There was suspicion for bacteremia as a source of thyroid infection, as one out of two blood cultures grew* Staphylococcus aureus.* While this positive blood culture could have been due to blood contamination with skin flora at the time of collection, her history of intravenous drug use and presence of skin abscesses made bacteremia more likely. In those with a history of IVDU, there is a possibility for bacteria and fungi on the surface of the skin, within an unsterilized syringe, or from a contaminated drug, to be introduced into the blood stream when a patient accesses the intravenous space in a nonsterile manner, leading to bacteremia. Chronic intravenous drug users often are colonized with* S. aureus *at a rate higher than those without IVDU [7].

Further hospital work-up showed no evidence of endocarditis or immunocompromisation, although it did show evidence of hepatitis C infection albeit with no evidence to suggest acute liver failure. Thyroid studies showed low TSH and high level of free T4 and free T3, indicating hyperthyroidism. She was subsequently started on propranolol with improvement of symptoms. Thyroid scintigraphy was not performed during inpatient hospitalization as the patient's symptoms began to improve with treatment of her infectious process. The patient was lost to follow-up after discharge from her inpatient hospitalization; therefore, no information regarding the remainder of her clinical course or further thyroid function is available.

While AST is a rare condition, it is important to recognize it as it can be fatal, with some estimating a 12% or higher mortality rate [3]. Fatal case reports have shown rupture of AST causing airway complication [8] leading to significant morbidity. Other complications of AST include vocal cord paralysis and permanent damage to the thyroid gland causing chronic hypothyroidism [3]. Treatment of AST is usually systemic antibiotic based on the specific causal organism. CT-guided percutaneous drainage has shown to be effective in some cases. In children with piriform sinus fistulae, ablation of the fistula is often necessary to prevent recurrent infection [3].

## Figures and Tables

**Figure 2:**